# A System of Medicine

**Published:** 1897-12

**Authors:** 


**A System of Medicine.** By Many Writers. Edited by Thomas Clifford Allbutt, M.D., F.R.S. Vol. III. Pp. xii., 1001. London: Macmillan and Co., Limited. 1897.

Two hundred and fifty pages of this volume deal with general diseases of obscure causation, including the various varieties of rheumatism, gout, diabetes, and lardaceous disease, while the other three-fourths deals with diseases of alimentation and excretion. The names of the authors are a sufficient guarantee of the value of the various articles. We greatly regret the cause which prevented Dr. Kent Spender from completing his excellent account of rheumatoid arthritis, and that the premature decease of Dr. Ralfe should have given his article on diabetes insipidus an especial value. To the former article Dr. Archibald E. Garrod has added various contributions, not the least important of which is that on the bacterial investigation of the fluid from the diseased joints, in which he mentions the observations of Ballantyne (it should be Bannatyne), Wohlmann and Blaxall.[1] He concludes this note by the remark, that in the light of these discoveries "the prevalent views as to the pathology of this disease will have to be largely revised." Of the special symptoms described by Dr. Spender, there are two which deserve especial attention, inasmuch as Dr. Spender's views thereon have not yet been altogether incorporated in the text-books. The changes in the circulation have been much overlooked: they are "frequent and important. Many cases of rheumatoid arthritis, rapid and crippling in their march, are characterised almost from the beginning by increased rapidity of the heart's action. The pulse-rate may go up gradually to 90 or 100, and remain so for years. . . . In 80 per cent. of the cases the pulse-rate was found to be more than 90." The occurrence of abnormal pigmentation of the skin is another feature on which Dr. Spender has been wont to rely: "The symptom was obvious and even obtrusive. . . . Concentrated in patches more or less large, the pigment assumes many hues and affects many parts of the body. Across the forehead it spreads as a light bronze smear, or like a dash of chloasma; over the temporal fossa on each side the tint may be deeper."[2] In cases of arthritis of doubtful character the rapid pulse and growing melasma, together with the cold and sweating hands, or neuralgic twinges in the upper and lower limbs, will be of material aid in diagnosis, at a time when the common failure to recognise the disease in its early and tractable stage is easily explained. The chapter on treatment has been added by Dr. Archibald E. Garrod, who very wisely states "that no greater mistake can be made than to treat the sufferer from rheumatoid arthritis as if he were an ordinary gouty subject."

The chapter on rheumatoid arthritis in children, by Dr. G. F. Still, calls attention to one especial variety of the disease, differing from the ordinary form as seen in adults, and from cases of chronic fibrous rheumatism; it may be defined "as a chronic progressive enlargement of joints, associated with enlargement of lymphatic glands and spleen."

Of the other rheumatic conditions, the article on acute rheumatism is the work of Dr. W. S. Church; but a special chapter on the acute rheumatism of childhood is added by Dr. W. B. Cheadle. An excellent drawing of the subcutaneous nodules in a grave form, with numerous large nodules, is given, and the microscopic characters of the nodules is also well illustrated. "They are seen to consist of nuclear growth in all

[1] *Lancet*, 1896, i. 1120; *Bristol M.-Chir. J.*, 1896, xiv. 123.
[2] See *Osteo-Arthritis*, by J. K. Spender, 1889.

stages of transformation into fibrous tissue—a proliferation analogous to that which is seen in the interstitial framework of the liver, or of the kidney, in cirrhosis. The connection of these nodules with rheumatism and rheumatoid arthritis is apparently absolute. So far as is known they own no other origin or relation." The grave significance of these nodules has been frequently commented upon, and it is stated that "they indicate concurrent and usually progressive cardiac disease. . . . In the acute rheumatism of children especially they have this ominous association with progressive endocarditis and pericarditis of the most serious kind." It is further pointed out that just as these nodules appear and rapidly disappear, so the concurrent proliferation and cell infiltration of the endocardium may undergo similar and rapid changes in valves and chords, accounting for the fact that the cardiac symptoms are sometimes so evanescent in character.

The chapters on chronic rheumatism, muscular rheumatism, and gonorrhœal rheumatism are written by Dr. A. E. Garrod, who describes the last named as a "definite specific disorder, comparable to true rheumatism, but essentially different from it." He further adds: "It must now be regarded as an established fact that, in some cases at any rate, there are present in the fluid obtained from the joints both of infants and adults, in the blood, in the pleural exudation and the cardiac vegetations, diplococci which present the characteristic shape of the gonococcus"; and further, "one can no longer doubt the dependence of the above-mentioned lesions upon the dissemination of the gonococcus." Does not this fact tend to favour the view that true rheumatism may also be due to some kind of organism which has not yet been described, and further, that the acute phases of polyarthritis (rheumatoid arthritis) are due to the presence of some such micro-organism as has been recently described?

The chapter on diabetes mellitus, by Dr. Robert Saundby, gives a good summary of the latest views on the pathology of this interesting malady; but we notice that the author hesitates to adopt the views so emphatically laid down by Dr. Pavy in opposition to the commonly accepted views of Claude Bernard. As regards the restriction of diet, Dr. Saundby's rule is to "permit each patient to enjoy the maximum amount of carbohydrates that he can assimilate."

Dr. Charles Henry Ralfe's contribution on diabetes insipidus describes not only the ordinary form of the disease, which he calls hydruria, but opens up other interesting and collateral topics—azoturia, phosphaturia, and baruria. It is somewhat astonishing to find that of the cases of phosphaturia which he has been able to trace, six have died of phthisis, three passed into diabetes mellitus, and one into rapid diabetes insipidus.

Passing on to the diseases of alimentation and excretion, we wonder how it comes to pass that an excellent account of shock and collapse by Dr. Louis Cobbett should be interposed as a sort of preliminary to diseases of the mouth, and why this latter should be followed by diseases of the œsophagus, with no mention of the important parts between. Of the various monographs on diseases of the stomach, we may especially mention that by the editor on his well-known and interesting topic, the neuroses of the stomach and other parts of the abdomen. Both here, and in the following chapter on dilatation of the stomach, he alludes to the question of gastroptosis, so intimately associated with neuralgia. A separate chapter on enteroptosis is written by Mr. Frederick Treves, who, after a consideration of the manner in which the chief viscera are suspended or supported, proceeds to deal with the displacement of the several viscera, then discusses "general ptosis of the abdominal organs, and that medley of symptoms which has been described under the title of Glénard's Disease." He remarks: "It is probable that among feeble and unhealthy women who lead inactive lives and who suffer from dyspepsia and constipation, this ptosis of the viscera is more common than is supposed," and that "in all civilised communities there is a pallid host of feeble folk who are flabby both in body and in mind, who can take little share in the business of the world, and who assume more or less pathetically the pose of chronic exhaustion." As regards the diagnosis of these displacements, we have found the combined auscultation and percussion method to be of much more service than is indicated by the editor.

Of the various chapters on the diseases of the peritoneum and of the bowels, we may mention that by Dr. Hale White on membranous colitis, which is much more complete than the usual descriptions of this by no means rare but very intractable malady: it is advised that in extreme cases the diseased bowel should be given complete rest, by the performance of colotomy on the right side.

This volume, like those preceding it, is a mine of wealth, deserving prolonged and careful study.

